# Direct in-water radiation dose measurements using Cherenkov emission corrected signals from polarization imaging for a clinical radiotherapy application

**DOI:** 10.1038/s41598-022-12672-w

**Published:** 2022-06-10

**Authors:** Émily Cloutier, Luc Beaulieu, Louis Archambault

**Affiliations:** 1 Centre Intégré de cancérologie and Axe Oncologie du CRCHU de Québec - Université Laval, CHU de Québec - Université Laval, Québec, G1R 2J6 Canada; 2 Département de physique, de génie physique et d’optique, et Centre de recherche sur le cancer, Université Laval, Québec, G1V 0A6 Canada

**Keywords:** Medical research, Energy science and technology, Oncology, Techniques and instrumentation

## Abstract

Cherenkov emission (CE) is a visible blueish light emitted in water mediums irradiated by most radiotherapy treatment beams. However, CE is produced anisotropically which currently imposes a geometrical constraint uncertainty for dose measurements. In this work, polarization imaging is proposed and described as a method enabling precise 2D dose measurements using CE. CE produced in a water tank is imaged from four polarization angles using a camera coupled to a rotating polarizer. Using Malus’ law, the polarized component of CE is isolated and corrected with Monte Carlo calculated CE polar and azimuthal angular distributions. Projected dose measurements resulting from polarization-corrected CE are compared to equivalent radiochromic film measurements. Overall, agreement between polarized corrected CE signal and films measurements is found to be within 3%, for projected percent depth dose (PPDD) and profiles at the different tested energies ($\gamma$: 6 and $18\,\hbox {MV}$, e$^{-}$: 6 and 18$\,\hbox {MeV}$). In comparison, raw Cherenkov emission presented deviations up 60% for electron beam PPDDs and 20% for photon beams PPDDs. Finally, a degree of linear polarization between 29% and 47% was measured for CE in comparison to $0.2\pm 0.3$% for scintillation. Hence, polarization imaging is found to be a promising and powerful method for improved radio-luminescent dose measurements with possible extensions to signal separation.

## Introduction

The more recent treatment modalities in radiation therapy now more often make use of small fields, very high dose rates (i.e. FLASH) and magnetic fields, which are non-standard, and pushes against the limits of radiation therapy technologies, and pose new dose measurement challenges. These modalities highlight the need for high-spatial resolution, real time, water-equivalent dosimeters to measure accurately and rigorously the doses delivered^1^. Given their intrensic qualities, luminescent dosimeters may play a key role in the development of modern radiotherapy^2^. In fact, luminescent dosimeters, such as Cherenkov and scintillation detectors, are recognized for their high spatial resolution, water-equivalence and dose rate independence over a broad range of therapeutic energies^3^. Moreover, these detectors have the potential of near real-time measurement because of the radio-luminescence being emitted within nanoseconds^4,5^.

Cherenkov radiation is a visible blueish light emitted in any dielectric mediums when a charged particle travel at a speed greater than that of light in that medium^6^. Given the refractive index of water ($n = 1.33$), Cherenkov radiation will be emitted by most therapeutic beams ($E_{\min } > 264\,\hbox {keV}$). Thus, it has the potential for perturbation-free in-water direct dose measurement (2D and 3D)^7^. Indeed, Cherenkov radiation has been demonstrated to be a useful tool for monitoring beam shapes, in particular for breast cancer radiotherapy^8,9^. However, due to its production mechanism, Cherenkov radiation is directional which imposes a source-phantom-detector geometrical dependency that is hard to account for when measuring dose^10,11^. In fact, the Cherenkov signal angular distribution depends on the beam energy spectra and direction which vary with the beam orientation and tissue attenuation. Because of Cherenkov anisotropic emission, converting a Cherenkov signal into a dose measurement is difficult. Before now, the only possible way to determine an absorbed dose from a Cherenkov reading was with a priori knowledge of the source-phantom-detector geometry or the use of telecentric lenses^12^ that limit measurements to smaller fields of view. As a result, Cherenkov emission is often treated as a spurious signal for which many removal techniques were proposed^3,13^. To tackle this challenge, some investigated the use of Monte Carlo simulations to calculate Cherenkov-to-dose conversion factors^14,15^. Others studied the potential of converting Cherenkov emission into an isotropic signal using a quinine fluorophore addition to water^7,16^. While Cherenkov spectra and intensity are well known, fewer work have looked into characterizing and using the Cherenkov radiation polarization state despite the anticipated benefits in using additional information. For example, the use of a set of two perpendicular polarizers enabled Cherenkov signal subtraction from fluorescence for carbon ion irradiation^17^.

Polarization imaging has proven benefits for detecting stress, surface roughness and other physical properties that cannot be detected using conventional imaging. Given the intrinsic polarization of Cherenkov emission, polarization imaging has potential benefits for dose measurements in radiotherapy that have not yet been exploited. Recently, a letter was published that presented promising results when using polarization imaging to correct the Cherenkov emission anisotropy which enabled accurate dose measurements^18^. The method takes advantage of polarization imaging to isolate the polarized contribution of the Cherenkov signal. Since the Cherenkov emission polarization vector is related to the Cherenkov emission cone, and thus its direction, Cherenkov radiation anisotropy can be corrected using the additional information provided by polarization imaging. Given these promising results, this paper further investigates Cherenkov emission dose measurements by extending results to other photon and electron beam energies. Moreover, this paper presents a detailed study of Cherenkov radiation angular distributions. To that effect, Monte Carlo simulations have been conducted to detail the Cherenkov signal angular distribution at different depths and off-axis positions. The degree and angle of linear polarization of Cherenkov and scintillation signals have additionally been characterized. The polarization signature of radioluminescent elements, together with the developed polarization imaging formalism, is a promising avenue for differentiating different radioluminescent sources in a single detector.

## Results

### Cerenkov angular distributions

**Figure 1:**
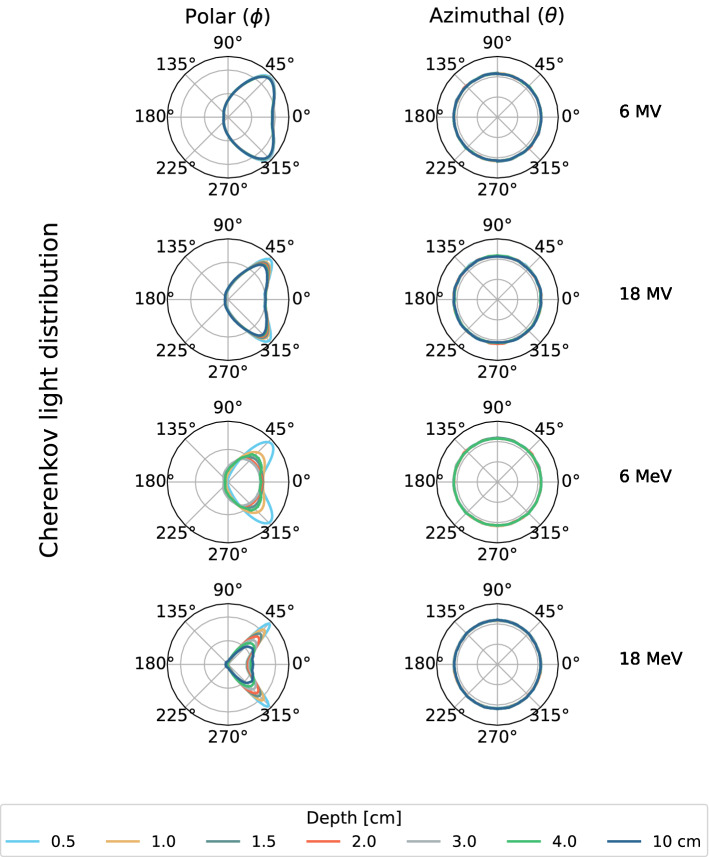
Cherenkov signal polar and azimuthal distributions extracted from different depths along the beam central axis using Monte Carlo simulations. The distributions were acquired for 6$\,\hbox {MV}$, 18$\,\hbox {MV}$, 6$\,\hbox {MeV}$ and 18$\,\hbox {MeV}$ beams. Data at 10 cm depth was not extracted for the 6$\,\hbox {MeV}$ beam because too little dose reaches that depth.

Cherenkov emission angulation was recorded from simulations at different depths along the beam central axis and on the beam profile at depth of maximal dose ($d_{\max }$). Figure 1 presents Cherenkov signal angular distributions, decoupled into polar and azimuthal components, for all irradiation beams tested (6$\,\hbox {MV}$, 18$\,\hbox {MV}$, 6$\,\hbox {MeV}$ and 18$\,\hbox {MeV}$) at different depths, obtained from Monte Carlo. For all beam energies, a maximal amount of signal is found at a polar angle of approximately 41$^{\circ }$. Measuring projected percent depth dose up to a depth of 17 cm corresponds to sampling these distributions from 80$^{\circ }$ to 100$^{\circ }$, approximately (this, of course, depends on the position of the camera in relation to the water tank). From Fig. 1, it is to be noted that similar Cherenkov signal angular distributions resulting from photon beam irradiations are obtained following $d_{\max }$, which is not the case for electron beams. Thus while a single correction function could be used for photon beams, electron correction functions need to take into account the depth at which a measurement is made. Overall, azimuthal distributions remained constant at all depths and for all energies.

**Figure 2:**
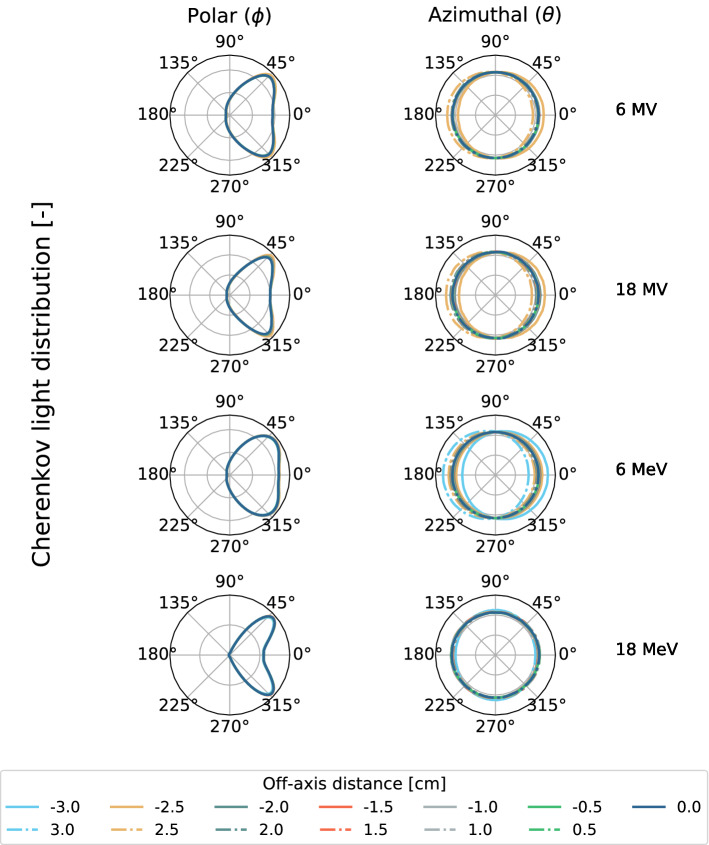
Cherenkov signal polar and azimuthal distributions extracted from different off-axis positions at the depth of maximum dose ($d_{\max }$). The distributions were acquired from 6$\,\hbox {MV}$, 18$\,\hbox {MV}$, 6$\,\hbox {MeV}$ and 18$\,\hbox {MeV}$ beams. ± 3 cm curves are not plotted for photon beams as these positions are larger than the beam size.

Figure 2 presents Cherenkov signal angular distributions scored from different off-axis distances at the depth of maximal dose, using Monte Carlo. In that case, polar distributions are fairly constant for all off-axis distances whereas the azimuthal distribution evolves. This is attributed to the loss of electronic equilibrium in the beam penumbra that favors electron propagation towards the edges of the beam. When measuring profiles with a camera, this corresponds to measuring angles from 86.5$^{\circ }$ to 93.5$^{\circ }$ approximately. In that regard, the azimuthal distribution predicts signal variations lower than 0.1%. Hence, geometrical signal variations are expected to be minimized when measuring profiles, especially for small fields.

### Dose measurements and corrections

**Figure 3:**
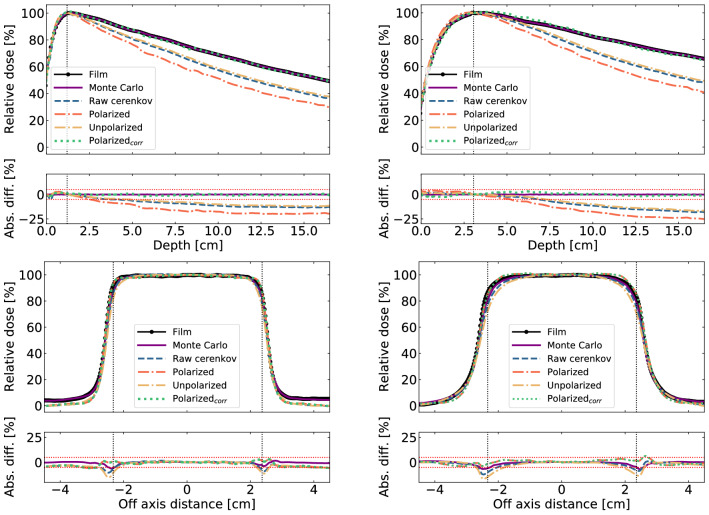
Photon beams projected percent depth dose (top) and profiles (bottom) sum over the thickness of the water tank and compared with dose measured from radiochromic films. Monte Carlo results are also presented for comparison. Vertical dotted lines indicate the position of maximum dose, while horizontal red dotted lines indicate a $\pm 5$% difference region. Left and right figures respectively present results from 6$\,\hbox {MV}$ and 18$\,\hbox {MV}$ beams.

**Figure 4:**
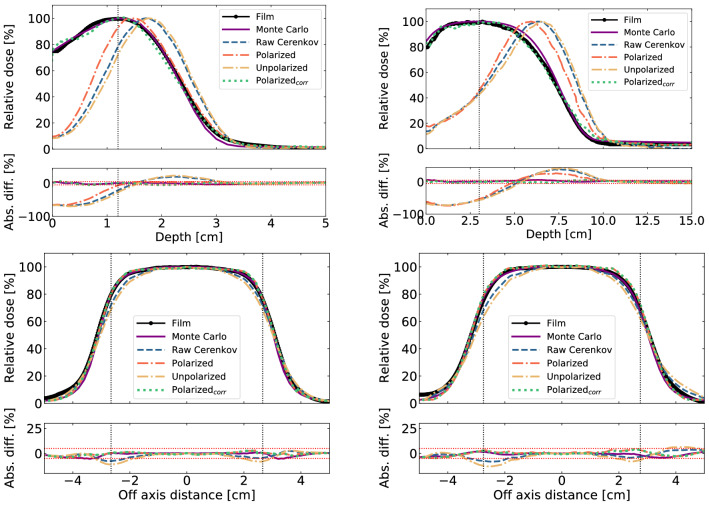
Electron beams projected percent depth dose (top) and profiles (bottom) compared with dose measured from radiochromic films. Monte Carlo results are also presented for comparison. Vertical dotted lines indicate the position of maximum dose, while horizontal red dotted lines indicate a $\pm 5$% difference region.Left and right figures respectively present results from 6$\,\hbox {MeV}$ and 18$\,\hbox {MeV}$ beams.

Figures 3 and 4 present projected profiles and PPDD measurements resulting from Cherenkov emission polarization dose imaging. For all measurements the absolute difference between films and Cherenkov signals is plotted and defined as: Cherenkov−film. Projected profiles and PPDD extracted from the Monte Carlo simulations are also presented for reference.

#### Photon beams

The top panel of Fig. 3 presents projected percent depth doses obtained at 6$\,\hbox {MV}$ and 18$\,\hbox {MV}$. On each figure is plotted the skewed polarized and randomly polarized PPDD obtained from Malus analysis. For comparison purposes, the raw Cherenkov response, obtained from measurements without polarizer, is plotted. Finally, dosimetric film measurements are shown against the resulting polarized signal corrected for directionality using Monte Carlo derived $\mathcal {C_\phi }(x, y)$ and $\mathcal {C_\theta }(x, y)$ correction functions. It is to be noted that film data are also projected data obtained from summation along the axis matching the optical axis. Corrected polarized Cherenkov signal presents mean ± standard deviation from films of $0\pm 2\%$ and $0\pm 2\%$ at 6$\,\hbox {MV}$ and 18$\,\hbox {MV}$ respectively. This is better than raw Cherenkov emission measurements reaching errors up to 20% for the 18$\,\hbox {MV}$ beam.

Similarly, the bottom panel of Fig. 3 presents projected profiles drawn at the depth of the maximum dose, i.e. 1.2 cm at 6$\,\hbox {MV}$ and 3.05 cm at 18$\,\hbox {MV}$. On the beam central axis, mean deviations from films of $0.7\pm 0.7\%$ and $1.5\pm 0.9\%$ at 6$\,\hbox {MV}$ and 18$\,\hbox {MV}$ were measured. The corrected signal presents greater discrepancies in the penumbra region where electronic equilibrium is lost for the 18$\,\hbox {MV}$ beam. Regarding the field sizes, the corrected signal results in a field size of $5.13\pm 0.03$ cm which compares to the $5.1\pm 0.1$ predicted from the film at 6$\,\hbox {MV}$. $5.22\pm 0.03$ cm and $5.0\pm 0.1$ are respectively obtained for 18$\,\hbox {MV}$ measurements.

#### Electron beams

Figure 4 top panel presents PPDD obtained for 6$\,\hbox {MeV}$ and 18$\,\hbox {MeV}$ electron beams. As for photon beam measurements, raw Cherenkov signal, unpolarized, polarized and polarized corrected signals are plotted against the expected doses extracted from film measurements. Unlike the photon beams correction which did not require taking into account the variation of Cherenkov emission angular distribution with depth, electron measurements did have to take this phenomenon into account. The correction function was found especially necessary in the first centimetres where raw Cherenkov emission largely underestimates dose: Cherenkov light’s angular distribution reduces the signal collected by the camera at these positions. Polarized corrected Cherenkov signals present differences with film measurements of $-1\pm 2\%$ (6$\,\hbox {MeV}$) and $0.9\pm 2\%$ (18$\,\hbox {MeV}$).

Figure 4 bottom panel presents projected profiles for both electron beams. Similarly to photon beam projected profile measurements, polarized corrected signal presents more deviations from film in the beam penumbra. Still, differences are lower than the signal obtained from raw Cherenkov emission measurements. Mean differences using corrected polarized Cherenkov signal of $0.9\pm 0.6\%$ and $0.8\pm 0.6\%$ are obtained on the beam central axis at 6$\,\hbox {MeV}$ and 18$\,\hbox {MeV}$. On the beam penumbra, those differences were of $1.5\pm 0.4\%$ and $1.2\pm 0.8\%$.

### Degree and angle of linear polarization

**Table 1:** Comparison of the mean degree of linear polarization (DoLP) of scintillation and Cherenkov emission. DoLP = $\hbox {I}_{\text {pol}}/\hbox {I}_{\text {tot}}\times 100$.

	Cerenkov				Scintillation
	6$\,\hbox {MeV}$	18$\,\hbox {MeV}$	6$\,\hbox {MV}$	18$\,\hbox {MV}$	
Mean ± STD [%]	42±4	47±3	29±1	33±1	0.2±0.3

**Figure 5:**
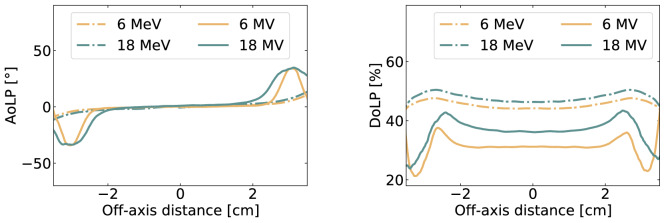
Angle (left) and degree (right) of linear polarization along profiles for the 6 MeV, 18 MeV, 6 MV and 18 MV beams. The profiles are taken at the depth of maximum dose for each energy.

Table 1 presents a comparison of the mean DoLP of Cerenkov radiation versus scintillation, extracted from the beam central region. As expected, the scintillation signal can be treated as non-polarized contrary to Cherenkov light that presents a degree of linear polarization from 29% to 47% depending on the beam energy. Figure 5 presents the variations of the angle and degree of linear polarization along the profile at depth of maximum dose for each beam energy. It was found that the Cherenkov radiation DoLP increased in the beam penumbra. We hypothesized that this is, in part, caused by perspective imposed by the camera. Polarized signal is attributed to signal polarization contributions that are not averaged into an unpolarized signal, which could be reinforced by perspective. Here, one should keep in mind that Cherenkov emission, by nature, should be 100% polarized. However, the signal reaching the cameras results from different contributions, resulting for example from the water tank thickness as well as the different electrons trajectories, which both averages into a partly polarized signal. In that sense, parallax may contribute to higher DoLP on the beam edges. Finally, as presented on the left panel of Fig. 5, the angle of polarization on the beam central axis is around 0$^{\circ }$ for all energies. However, the AoLP changes in the beam penumbra where is reaches 35$^{\circ }$ for the photon beams.

## Discussion

Cherenkov radiation angular distribution, which depends on the beam energy and direction, is currently limiting applications for accurate dose measurements. Because of it, the signal collected strongly depends on the source-phantom-detector geometry rather than just the absorbed dose. In this work, both polar and azimuthal angular distributions were recorded to quantify and correct this geometrical limitation. First, it was found that Cherenkov radiation exhibits polar angular distributions peaked at 41$^{\circ }$ for photon and electrons beams. This means that orienting a photodetector at 41$^{\circ }$ from the beam will maximize the signal collected. These results agree with the ones from Zlateva et al.^14,15^. Moreover, with electron beams, polar angular distributions vary with depth which further complicates dose measurements. This was shown in Fig. 4 where the raw Cherenkov signal is minimized in the first centimeters. Cherenkov angular distributions are closely related to the electron scattering angles and energy in the medium, as seen in equation 4. In Fig. 4 the expected electron scattering behavior is observed, which is primarily in the forward direction, but becomes more isotropic at depth. Cherenkov angular distributions generated from photon beams irradiations are more constant because the electron energy fluence is more constant with depth due to the indirect ionization from photons. This explains how a single correction function is possible for photon beams, but not for electron beams.

Azimuthal distributions are fairly constant at all points in space, which reduces the need for angular corrections. This was to be expected from previous work where projected profiles measured from Cherenkov signal agreed with the expected dose^11,16^. Still, better agreement was found in this work with polarized Cherenkov emission than raw Cherenkov signal. The higher deviations from film measurements were found in the beam penumbra and umbra. In the beam penumbra, an overestimation of the dose is possible due to perspective from the imaging system. In fact, a higher degree of polarization is found in that region. Polarized signal is attributed to signal polarization contributions that are not averaged into an unpolarized signal. Averaging is mainly attributed to the fact that a measurement corresponds to the signal summed over the water tank thickness. Perspective, combined with the high dose gradient toward the edges could generate a shoulder dose overestimation as the contributions from the plane closer to the camera are not averaged by the ones farther on the optical axis. In the beam umbra, dose underestimation, especially for lower energies (6$\,\hbox {MV}$ and 6$\,\hbox {MeV}$), is attributed to the dose being predominantly deposited by electrons below the Cherenkov emission production threshold in that region.

Polarization imaging of a scintillator validated that scintillation is unpolarized, unlike Cherenkov radiation. Still, Cherenkov radiation DoLP below 100% were measured even if Cherenkov emission is expected to be entirely generated with a linear polarization. However, measurements of the DoLP are conducted in a region were signal is summed with depth. In that case, contributions from different polarization angles are averaged and appear as unpolarized signals. Moreover, fluorescence, the water scintillation signal, may also contribute to the unpolarized signal^19^ since, as demonstrated, scintillation can be considered as unpolarized. Hence, polarization imaging would be an effective method to isolate scintillation from Cherenkov emission, using the DoLP. Similarly, Yamamoto and al. pursued promising Cherenkov signal removal using a set of two perpendicular polarizers for carbon-ion irradiation^17^. Given the results obtained from our AoLP analysis, that approach method should work on the beam central axis where Cherenkov radiation is mostly emitted at 0$^{\circ }$. However, we demonstrated that the angle of polarization changes in the beam penumbra and reaches 35$^{\circ }$. Hence, complete AoLP measurement using polarization imaging (all four angles) could ensure a more robust Cherenkov emission removal in measurement conditions where the AoLP varies.

Integration times of 30 seconds were used to ensure sufficient signal-to-noise ratio and reduce measurement uncertainties. In fact, the signal output from Cherenkov emission is lower than scintillation and adding a polarizer before the camera further cuts the initial signal by at least 50%: an unpolarized signal will be attenuated by half. Given the resulting DoLP and AoLP of Cherenkov radiation, some polarizer configurations decreased the Cherenkov signal to 30% of its initial, raw value. The expected signal reduction $I/I_0$ can be estimated using equation 3. The reduction depends on the amount of polarized signal, and the difference between the signal’s angle of linear polarization and the polarizer axis. To reduce the integration time, timed-gated intensified cameras were found suitable for Cherenkov dosimetry^20,21^. Triggering the acquisition on linac pulses and amplifying that signal increases the detectability and sensitivity of the system. Using such cameras enabled real-time dose measurements using Cherenkov signals^22^. Having a more sensitive detecting device would allow more precise measurements while allowing the camera to be placed farther from the irradiation beam. This would reduce perspective^23^ as well as stray radiation noise^24^. Moreover, acquisition could be optimized by using a sensor measuring simultaneously the polarization from four different transmission axis. Such sensors, called wire-grid arrays, have recently been made commercially available^25^. However, these sensors are currently implemented on non-cooled CMOS which are too noisy for polarized Cherenkov signal measurements.

The main limitation of the current method is its reliance on predetermined Monte Carlo calculated correction coefficients. However, the correction coefficients describe the Cherenkov emission directionality, which is not related directly to the dose. Hence, because the correction coefficient do not result from a circular logic, they are expected to be more robust to beam differences than previously used direct Cherenkov emission to dose correction coefficients. For example, the angular distributions for photon beams were fairly constant through the irradiated volume and varied only slightly from 6$\,\hbox {MV}$ to 18$\,\hbox {MV}$. Angular distributions are, however, more challenging for electron beam irradiations. Although it is not trivial, it would be conceivable that the correction coefficients could be extracted from additional experimental measurements or from a theoretical development. Attempts in that direction have previously been published using optical fibers, yet it was demonstrated to be challenging^26,27^.

Polarized Cherenkov signals enabled precise measurement directly from radiotherapy’s medium of reference: water. Thus, Cherenkov radiation carries the potential to directly visualize and measure dose distributions in water tanks already used in the clinics. The method presented is currently limited to 2D dose measurements corresponding to the sum the dose produced in the water tank along the optical axis. However, previous work has shown 3D dose reconstruction from optical measurements being possible using acquisitions from orthogonal planes^28–31^. Hence, we expect that polarization imaging could be implemented into 3D dose measurement systems. In addition, the actual set-up could complement measurements performed with a conventional point detector in a water tank. Adding a polarized camera imaging to a commissioning water tank could increase the number of positions at which dose is measured simultaneously as well as guide measurements. For example, imaging Cherenkov emission from water tanks could help position conventional detectors in relation to the radiation field as the beam size and $d_{\max }$, for example, are accurately determined.

Benefits of polarization imaging, already demonstrated for machine vision applications, were evaluated in the context of Cherenkov emission-based dose measurements. It was found that polarization imaging can isolate the anisotropic signal contribution which can then be corrected using known angular Cherenkov emission distributions. Projected profile and depth dose measurements were performed using 6$\,\hbox {MV}$, 18$\,\hbox {MV}$, 6$\,\hbox {MeV}$ and 18$\,\hbox {MeV}$ medical linac beams. Overall polarized corrected Cherenkov signals presented mean differences within 3% of radiochromic films measurements in all irradiation conditions. The system is currently limited to 2D dose measurements that sum signals over the water tank thickness, and uses predetermined Monte Carlo correction coefficients. However, this is a first step towards direct in-water 3D precise Cherenkov dose distribution measurements.

## Methods

### Polarization imaging

Polarization is a key electromagnetic wave property related to the electric field oscillation direction. Using polarizers while measuring a signal can reveal the light polarization angle and the degree of polarization. The polarizer act as a filter that transmits light waves of a specific polarization state (angle) while blocking light waves of other polarization states. When measuring a polarized signal with a linear polarizer, the rise and fall in intensities transmitted is given by Malus’s law^32^:

1 $$I = I_{0} \cos ^{2} (\alpha _{0} - \alpha )$$

where $\alpha _{0} - \alpha$ is the angle between the light’s initial polarization direction and the polarizer transmission axis. The angle of polarization refers to the direction in which the electric field component of the electromagnetic wave oscillates while the degree of polarization describes the ratio of polarized signal over the total signal. A light beam composed of electromagnetic waves with randomly distributed electric field alignment is referred to a randomly polarized signal or unpolarized signals because contributions from every possible direction balances each other. By rotating a linear polarizer in between measurements, the degree of linear polarization (DoLP) and angle of linear polarization (AoLP) can be determined. An unpolarized light beam will result in a DoLP of 0% whereas a totally polarized signal will have a DoLP of 100%. We used a rotating polarizer to measure the Cherenkov signal from four angles forming two orthogonal pairs [0$^{\circ }$, 45$^{\circ }$, 90$^{\circ }$, 135$^{\circ }$]. To account for unpolarized signal we added a DC signal component to equation 1. Malus’ law thus becomes:

2 $$I = I_{{pol}} \cos ^{2} (\alpha _{0} - \alpha ) + \cdot I_{{{\text{Unpol}}}}$$

Now in an imaging context, $I_{\text {pol}}$ and $I_{\text {Unpol}}$ have to be retrieved at each pixel. Taking the pixel values *I*(*i*, *j*) acquired at each polarizer angle $\alpha _{0}$, DoLP and AoLP were determined with a fit of equation 2:

3 $$I_{{ij}} (\alpha _{0} ) = I_{{{\text{pol}},ij}} \cos ^{2} (\alpha _{0} - \alpha ) + \cdot I_{{{\text{Unpol}},ij}} .$$

#### Polarized image acquisition

**Figure 6:**
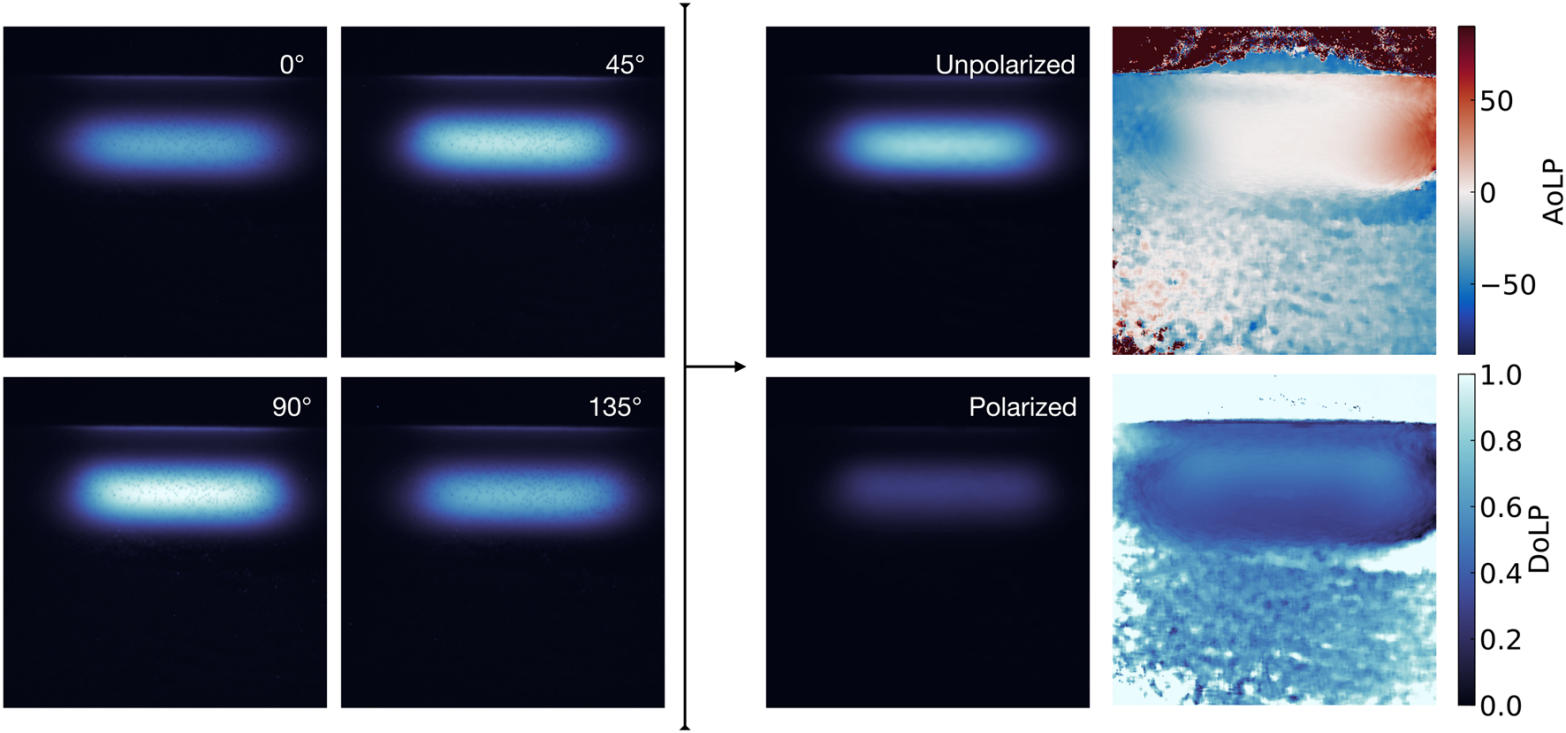
On the left are presented the four images acquired from the four polarization states (0$^{\circ }$, 45$^{\circ }$, 90$^{\circ }$, 135$^{\circ }$) for a 6$\,\hbox {MeV}$ beam. The right panel presents the resulting images following the polarization imaging formalism. The unpolarized and polarized portions of the signals are presented along with the degree and angle of linear polarization extracted from each pixel.

To collect the signal, a CCD camera (Atik 414EX; Atik Cameras, Norwich, United Kingdom) was placed on the treatment couch of a medical linac (Clinac iX, Varian, Palo Alto, USA) and used to image the Cherenkov signal produced in a $15\times 15\times 20\,\text {cm}^{3}$ water tank. The camera was set 50 cm from the water tank and coupled to a 12 mm variable focal length optical lens. The camera optical axis was aligned on the water tank center. The pixel to mm calibration was performed using a prior image of a chessboard pattern at the distance of interest. The resulting dose images have a $0.26\times 0.26$ mm$^2$ pixel size (array of 1391x1039 pixels). A linearly polarized filter (XP42-18; Edmund Optics, Barrington, NJ) was positioned in front of the camera and provided the measurements from the four polarizer angles. Hence, for each irradiation geometry, the signal at each pixel could be described by a set of four points acquired from the different polarizer orientations. Figure 6 presents an example of the dataset acquired from an electron beam irradiation and the resulting images that can be produced using equation 3.

Measurements without a polarizer in front of the camera were also acquired to compare polarized data with raw Cherenkov measurements. For each irradiation condition, ten signal images were acquired with an integration time of 30 s and treated with a median temporal filter to reduce transient noise^24^. Ten background frames were averaged and subtracted from the signal images. Frames were further flat field corrected using a uniform white emitter screen and a $\cos ^4 (\theta _{(i,j)} )$ fit as proposed by Robertson et al.^23^. Room ambient light was minimized by covering the set-up with black opaque blankets.

#### Cherenkov radiation

**Figure 7:**
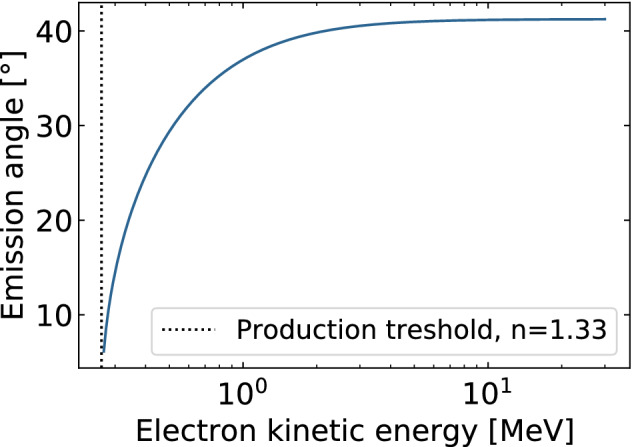
Cherenkov signal emission angle as function of a charged particle kinetic energy, an electron in that case, in water ($n = 1.33$).

Cherenkov light is emitted along a cone whose angle is determined by the velocity *v* of the charged particle and the refractive index *n* of the surrounding medium. The Cherenkov light direction $\theta$ and production threshold $E_{\min }$ are respectively given by:

4 $$\cos (\theta ) = c \cdot [vn]^{{ - 1}}$$

5 $$E_{{\min }} = m_{0} c^{2} \cdot \left[ ({1 - 1/n^{2} })^{-1/2} -1\right]$$

where *c* is the light speed constant, *E*_min_ is the relativistic kinetic energy and $m_0c^2$ is the charged particle relativistic mass energy. Hence, the angle at which a maximum of Cherenkov signal is to be collected depends on the beam’s charged particles direction and kinetic energy. Figure 7 presents the angle of Cherenkov emission as a function of an electron kinetic energy in water ($n = 1.33$). Thus, in water, Cerenkov is expected to be produced at a maximum of 41$^{\circ }$ from the particles path.

Cherenkov light is polarized as a result of the dipoles oscillation producing the signal. The polarization direction coincides with the average spin of the electron^33^ and is perpendicular to the cone emission, pointing away the particle’s path^34^.

### Dose measurements

**Figure 8:**
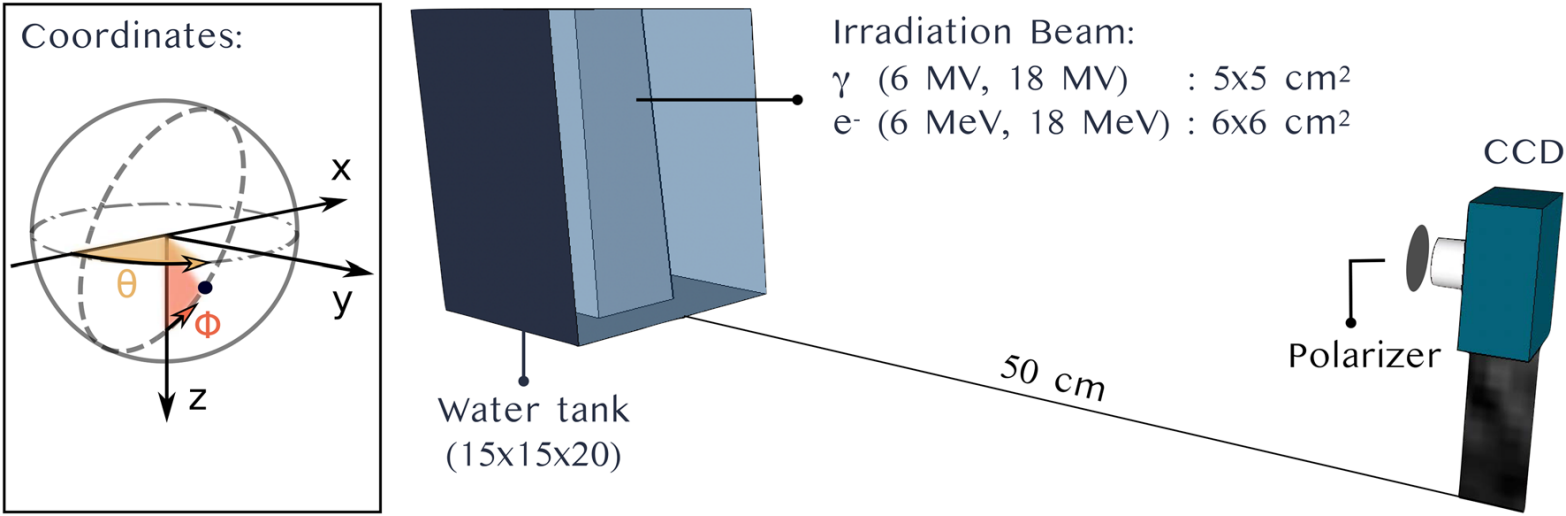
Representation of the measurement setup together with the irradiation conditions for PPDD and profile measurements. The setup image was created using SketchUp^35^.

Cherenkov emission was measured in a $15\times 15\times 20\,\hbox {cm}^3$ water tank irradiated with both photon and electron beams. The water tank was positioned at a source to surface distance (SSD) of 100 cm. Figure 8 summarizes the irradiation conditions and presents a representation of the water tank and irradiation beam. The resulting images correspond to the Cherenkov signal summed over the water tank thickness along the optical axis. As a result, projected dose distributions are measured^18^. For each set of measurements, projected percent depth dose (PPDD) and projected profiles at depth of maximum dose ($d_{\max }$) were extracted. In fact, The inner walls of the tank were covered with a thin opaque black film to minimize the collection of reflection signals.

Dose measurements were reproduced with gafchromic EBT3 films (Gafchromic EBT3, Ashland Inc., Covington, KY) for comparison. Film sheets were inserted in a solid water phantom mimicking the irradiation conditions for Cherenkov-based dose measurements. Dose was summed along the beam width, both for projected profile and PPDD measurements, similar to the Cherenkov imaging measurements.

#### Polarized corrected Cherenkov dose measurements

Using polarization imaging, we extracted the polarized Cherenkov signal from the total signal. We hypothesized that the polarized signal is proportional to the dose if the Cherenkov signal anisotropy is taken into account, that is when the geometrical signal dependency imposed by the position of the camera relative to each dose measurement position in the water tank is removed. Hence, Monte Carlo simulations using the Geant4 toolkit (v4.10.04)^36^ were performed to extract the polar and azimuthal distributions of Cherenkov radiation production. Geant4 was chosen for Cherenkov simulations because of its validated optical module whereas phase spaces were generated using the specifically developed BEAMnrc GUI from EGSnrc^37^. Hence, clinac $5\times 5\,\hbox {cm}^2$ (photons) and $6\times 6\,\hbox {cm}^2$ (electrons) phase spaces were generated using BEAMnrc. The component modules were produced per the available manufacturer descriptions on the www.myvarian.com website. Phase spaces were generated for 6$\,\hbox {MV}$, 18$\,\hbox {MV}$, 6$\,\hbox {MeV}$ and 18$\,\hbox {MeV}$ beams and irradiated a $15\times 15\times 20\,\hbox {cm}^3$ water tank at a 100 cm SSD, similar to the experimental measurements. The direction and position of each Cherenkov photon produced in the water tank were tallied to generate the polar $\theta$ and azimuthal $\phi$ angular distributions. Given these angular distributions describing the anticipated directionality of Cherenkov emission, the dose would be given by:

6 $$\begin{aligned} D(x, y) \propto \mathscr{C}_{\theta }(x, y) \cdot \mathscr{C}_{\phi }(x, y) \cdot I_{\text {pol}}(x, y) \end{aligned}$$

where $\mathscr {C}_{\theta }(x, y)$ and $\mathscr {C}_{\phi }(x, y)$ refer respectively to the polar and azimuthal angular Cherenkov emission dependency corrections. To better represent the collection efficiency of the CCD, only photons having $\theta > 0$ were scored for angular distribution analysis. Those distributions were used to correct skewed Cherenkov radiation dose distributions arising from the directionality of the signal. It was found that $\mathscr {C}_{\theta }(x, y)$ and $\mathscr {C}_{\phi }(x, y)$ were fairly constant in the electronic equilibrium region of photon beams. Hence, for depth dose measurements, only a single angular distribution acquired at the center of the water tank was used for corrections for both 6 and 18$\,\hbox {MV}$ beams. However, for photon beam profile measurements and all electron beam measurements, the angular distribution was shown to vary across the volume which required the sampling of the angular distributions at many positions in the volume.

### Degree of linear polarization

The Cherenkov emission degree of linear polarization was compared to that of a pure scintillation emission using an organic plastic scintillator. To do so, a $3\times 3\times 3\,\hbox {cm}^3$ scintillator (EJ212; Eljen technology, Sweetwater, TX) was irradiated by a 120 kVp orthovoltage beam (Xstrahl 200, Camberley, United Kingdom). The scintillator was imaged by the same camera-polarizer set-up as the one described for MV Cherenkov signal measurements. As 120 kV is below the Cherenkov emission threshold in polyvinyl toluene ($n = 1.58$, $E_{\min } = 149\,\hbox {keV}$), the signal is solely composed of scintillation. The resulting DoLP was extracted from a $1\times 1\,\hbox {cm}^2$ region of interest and compared to the one obtained from Cherenkov emission.

## Data Availability

All data needed to evaluate the conclusions in the paper are present in the paper. The data are available from the corresponding author, EC, upon reasonable request.
